# miR‐34a/BCL‐2 signaling axis contributes to apoptosis in MPP^+^‐induced SH‐SY5Y cells

**DOI:** 10.1002/mgg3.469

**Published:** 2018-09-16

**Authors:** Zahra Shanesazzade, Maryam Peymani, Kamran Ghaedi, Mohammad Hossein Nasr Esfahani

**Affiliations:** ^1^ Department of Biology, Faculty of Basic Sciences, Shahrekord Branch Islamic Azad University Shahrekord Iran; ^2^ Department of Biology, Faculty of Sciences University of Isfahan Isfahan Iran; ^3^ Department of Cellular Biotechnology, Cell Science Research Center Royan Institute for Biotechnology, ACECR Isfahan Iran

**Keywords:** *BCL‐2*, dopaminergic neurons, miR‐34a, Parkinson's disease, SH‐SY5Y

## Abstract

**Background:**

Parkinson's disease (PD) is a neurodegenerative disorder which mainly affects the elderly population of various societies. The main hallmark of this disease is the loss of dopaminergic (DA) neurons. So far, numerous studies have implied the role of microRNAs in fine‐tuning cellular processes including apoptosis. Studies have also shown that miR‐34a is mainly involved in age‐related disorders including Alzheimer's disease, and its expression is usually higher in the brain sample patients. Furthermore, the key role of miR‐34a in the expression of *BCL‐2,* and thus, in vitro and in vivo apoptosis has been revealed. miR‐34a/*BCL‐2* axis is therefore of critical importance in inducing or inhibiting apoptosis.

**Methods:**

In this study, human SH‐SY5Y cells were treated with MPP+ and the expression of miR‐34a and *BCL2* was assessed.

**Results:**

Our results also showed that treating human SH‐SY5Y neuronal cells using MPP^+^ to induce oxidative stress and apoptosis led to the upregulation of miR‐34a, as compared to the nontreated control group. Moreover, evaluating the expression level of *BCL‐2* in these cells indicated a contradictory pattern, as compared with miR‐34a. It was also revealed that the expression of *BCL‐2* was significantly decreased in MPP^+^‐treated cells, thereby confirming previous studies regarding a new concept. In this study, we show that miR‐34a/*BCL‐2* axis is directly correlated with oxidative stress and apoptosis in SH‐SY5Y cells as a model of DA neurons.

**Conclusion:**

miR‐34a and its target gene, *BCL‐2,* play a possible role in the induction of apoptosis in DA neurons, and therefore, they have a potential role in the pathogenesis of PD. Consequently, the therapeutic potential of miR‐34a could be considered in order to inhibit the progression of PD.

## 1 INTRODUCTION

Parkinson's disease (PD) is a progressive neurodegenerative disease of CNS and the second most common neurodegenerative disorder (De Rijk et al., 2000). PD shows multiple pathophysiological symptoms including oxidative stress, and mitochondrial and protein degradation dysfunctions, among other neuroanatomical derangements, in CNS, affecting 1% of the population over 50 (Golpich et al., 2015). However, the pathological hallmark of the disease is mainly the loss of dopaminergic (DA) neurons in *substantia nigra pars compacta* (SNc), corpus striatum, and brain cortex. DA neurons located in SNc send their projections to the dorsal striatum, which mainly regulates controlled movements, emotions, and complex behaviors. Progressive degeneration of these neural cells in human brains leads to motor symptoms in PD (Harraz, Dawson, & Dawson, 2011). So far, different studies have pinpointed various transcription factors and microRNAs (miRNAs) that play a key role in the development of DA neurons as well as pathogenicity of PD. However, we are still far from understanding the ample mechanism underlying the processes happening in these cells.

miRNAs are small noncoding RNAs which play important roles in the regulation of various cell processes such as proliferation, differentiation, and apoptosis (Bartel, 2004). The essential dependence of DA neurons on miRNAs has been revealed both in vitro and in vivo. A recent study has shown that the reduction in dicer in the ventral midbrain of aged mice leads to the decreased number of DA neurons, thereby indicating dicer and thus miRNA's crucial role in the maintenance of DA neurons (Chmielarz et al., 2017). Also, brain samples of PD patients have shown the great change of miRNAs expression, thereby suggesting the fundamental role of miRNAs in the course of the disease (Kim et al., 2007; Miñones‐Moyano et al., 2011). Various studies have explored deregulation of different miRNAs including the miR‐34 family, both in vitro and in vivo.

In human, the miR‐34 miRNA precursor family gives rise to three major mature miRNAs including miR‐34a, miR‐34b, and miR‐34c (Hermeking, 2010). Recent studies on the miRNA profiling of PD patients brain samples have determined the downregulation of miR‐34b and miR‐34c in different brain regions including *substantia nigra* and amygdala (Miñones‐Moyano et al., 2011). However, it is not specified whether the downregulation of these miRNAs is by virtue of DA neurons degeneration or their decrease in the surviving neurons.

Additionally, miR‐34a has been proposed as an acceptable biomarker in the plasma of the patients with neurodegenerative diseases (Li, Khanna, Li, & Wang, 2011). Moreover, miR‐34a is increased with age in mice cortex and hippocampus; also, studies have revealed miR‐34a and miR‐34c enrichment in the brain of Alzheimer's mice models (Zovoilis et al., 2011). Interestingly, a recent study has confirmed the potential of miR‐34a in PD therapy through inhibiting this miRNA and affecting Nrf2 pathway (Ba et al., 2015). Therefore, the miR‐34 family plays controversial roles and seems to be context‐dependent on the basis of the cell environment. *BCL‐2* is a member of an anti‐apoptotic family of genes usually located in the mitochondrial outer membrane (MOM) and prevents the activation of pro‐apoptotic proteins such as caspase‐9 (Pellegrini & Strasser, 2013). Interestingly, enhancing the *BCL‐2* expression in SH‐SY5Y cells using an anti‐PD drug called rasagiline has shown promising results in protecting neuronal degeneration in PD (Akao et al., 2002). Therefore, finding a possible miRNA‐*BCL2* correlation could be of paramount importance as various miRNAs are in different clinical trial phases to be used as drugs.

The aim of this study was to specifically evaluate the expression level of miR‐34a and the related target gene in the human SH‐SY5Y cell line; this was done as an in vitro model of DA neurons in which neurotoxicity was induced using MPP+. As various studies have shown the apoptotic effect of miR‐34a, its role in increasing P53 in stressed cells, and the miR‐34a role in aging‐related disorders such as Alzheimer's disease, we believe that miR‐34a plays a possible role in the apoptosis of DA neurons and pathogenesis of PD (Chang et al., 2007; Wang et al., 2009). The aim of this study was to suggest miR‐34a and one of its target genes, BCL‐2, as some novel miRNA‐gene axis involved in the DA neurons cell death and as a possible cause of pathogenesis in PD.

## 2 MATERIALS AND METHODS

### 2.1 Cell cultures and reagents

SH‐SY5Y cells were a DA neuron‐like cell line widely used as the in vitro model of DA neurons toxicity. SH‐SY5Y cells were cultured and maintained in DMEM/F12 supplemented with 10% FBS, 1% v/v penicillin–streptomycin, 1% v/v non‐essential amino acids (All from Gibco, USA), 1% v/v L‐glutamine (Sigma, USA), and a humidified atmosphere containing 5% CO_2_ at 37°C. The cells were passaged every 2 days to remain in the logarithmic phase during the experiments.

### 2.2 MTS [3‐(4, 5‐Dimethylthiazol‐2‐yl)‐2,5‐diphenyltetrazolium bromide] assay

#### 2.2.1 Selecting the optimum number of cell cultures

In order to find the optimal number of cell cultures, various numbers of SH‐SY5Y cells ranging from 625 to 16 × 10^4^ were seeded in 96‐well plates. Subsequently, 48 hr after seeding the cells, 20 µl of the MTS/PMS reagent was added to each well and cell proliferation was measured through the intensity of the absorbance using an ELISA reader.

#### 2.2.2 Selecting the optimal concentration of MPP^+^

SH‐SY5Y cells were seeded in 96‐well plates at a density of 2 × 104 cells per well. Cells were grown for 24 hr, and this was followed by adding various concentrations of MPP^+^ including (125, 250, 500, 750, 1,000, 1,500, 2,000, 3,000, 4,000, and 6,000 µM) in order to find the proper concentration. Cell viability was then determined using the MTS assay. Briefly, after incubation for 24 hr, they were incubated in a humidified, 5% CO_2_ incubator for 4 hr. The resulted formazan dye was dissolved in DMSO, and the absorbance was measured at 492 nm.

### 2.3 Flow cytometric analysis of apoptosis by Annexin‐V/PI

Briefly, in order to remove the medium, SH‐SY5Y cells were centrifuged at 10^3^×g. Next, cells were washed with PBS and stained with Annexin‐V (IQ products, USA) as well as PI in a binding buffer. Finally, the cells were analyzed on the Becton Dickinson flow cytometer (FACSCalibur, USA) using the CellQuest Pro software.

### 2.4 SH‐SY5Y cells treatment with MPP+

SH‐SY5Y cells were seeded at a density of 8 × 10^5^ in plates and treated with a concentration of 2,000 µM of MPP+ in order to evaluate the expression of miR‐34a and its target gene, *BCL*‐*2*.

### 2.5 RNA extraction

Total RNA (containing miRNA) was isolated from SH‐SY5Y using the TRizol reagent (Invitrogen, USA), and their quality was assessed by the 260/280 ratio using a spectrometer.

### 2.6 cDNA synthesis and real‐time PCR

Synthesis of cDNA for miR‐34a was performed using a “universal cDNA synthesis kit” (Exiqon, Denmark) based on a poly A tailing method, as specified by the manufacturer. Real‐time quantitative PCR (RT‐qPCR) products were conducted according to the standard protocols in an ABI PRISM 7,500 instrument (Applied Biosystems, USA). cDNA product was added to a master mix comprising 10 pmol/µl of miR‐34a predesigned primers (Exeqon, Denmark) and 2 U of SYBR premix Ex*Taq* II (TaKaRa, Japan) (Naghavian et al., 2015). The expression level of the RNU6 small nucleolar RNA was also evaluated as a reference miRNA to normalize the results. cDNA synthesis of *BCL‐*2 was conducted using the Revert Aid First Strand cDNA Synthesis Kit (TaKaRa, Japan), by utilizing the random hexamer primers. RT‐qPCR was performed using specific primer pairs in the ABI PRISM 7,500 instrument (Applied Biosystems, USA). The expression levels of *BCL‐*2 were normalized with *GAPDH* as the reference gene. All reactions were implemented in triplicate. RT‐qPCR primers for *BCL‐2* and *GAPDH* are listed in Table 1.

**Table 1:** RT‐qPCR primers for *BCL‐2* and *GAPDH*

mRNA	Primer name	Primer sequence (5′–3′)
*BCL‐2*	Forward primer	5′‐TGGAGAGTGCTGAAGATTGATG‐3′
	Reverse primer	5′‐AGTCTACTTCCTCTGTGATGTTG‐3′
*GAPDH*	Forward primer	5′‐CCACTCCTCCACCTTTGACG‐3′
	Reverse primer	5′‐CCACCACCCTGTTGCTGTAG‐3′

### 2.7 Statistical analysis

All statistical tests were carried out using SPSS (version 20) and analyzed by the statistical independent sample *t* test. Data were presented as mean ± *SEM* and considered significant at *p* < 0.05.

### 2.8 Molecular signaling pathway enrichment analysis

In order to conduct the molecular enrichment analysis on miR‐34a targetome and its target gene *BCL‐*2, and to find the most related signaling pathway and target gene in which miR‐34a might be involved, Targetscan and miRTarbase databases were used to find the possible target genes of miR‐34a. Moreover, miR‐34a targetomes were assigned to KEGG and PANTHER databases to categorize the most applicable pathways, molecular networks, and, thus, target genes of miR‐34a.

## 3 RESULTS

### 3.1 Inducing oxidative stress on SH‐SY5Y cells using MPP+

Generally, the high number of cells inhibits cell ability to proliferate, while the low number of them lacks the necessary cell communications to develop and proliferate. Therefore, the first step was to find the proper number of cells which had to be seeded in each well in order to see the sheer effect of oxidative stress on the cell proliferation and apoptosis. Our results showed that at the concentration of 2 × 10^4^ cells per well, SH‐SY5Y cells had the tolerable and adequate proliferation (The supplementary Figure 1a). Hereafter, 2 × 10^4^ cells were tested for different concentrations of MPP^+^ in order to examine their cytotoxic effects. The MTS assay for cell viability revealed that among various concentrations of MPP^+^, the concentration of 2,000 µM showed 50% cell death rate of SH‐SY5Y after 24 hr (the supplementary Figure 1b). Therefore, this concentration was selected to induce the neurotoxicity in the cells for the following experiments.

**Figure 1:**
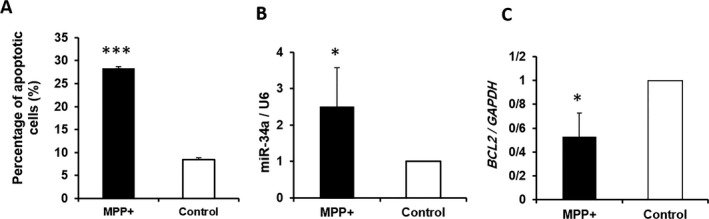
MPP^+^ **‐**mediated apoptosis of SH‐SY5Y cells. (a) Quantification of flow cytometry data shows apoptosis in MPP^+^‐treated cells, as compared to the nontreated control group. (b) Upregulation of miR‐34a in MPP^+^‐treated cells, as compared to the control group. miRNA results are normalized to U6 as the reference gene. (c) Downregulation of *BCL‐2* in MPP^+^‐treated cells, as compared to the control group. Results are normalized relative to *GAPDH* expression (*indicates *p* < 0.05, and ***is representing *p* < 0.001, relative to the control).

### 3.2 Investigating MPP+‐mediated apoptosis through Annexin‐V/PI

The rate of apoptosis was analyzed using Annexin‐V/PI by the flow cytometry analysis. As can be seen in Figure 1a, SH‐SY5Y cells treated with 2,000 µM MPP^+^ concentration showed a greater percentage of apoptotic cells with 2,000 µM of MPP^+^, as compared with the nontreated control group (Figure 1A).

### 3.3 Upregulation of miR‐34a in SH ‐SY5Y cells undergoing oxidative stress

Analysis of miR‐34a expression indicated that miR‐34a displayed an elevated expression in MPP^+^‐treated cells, as compared to the nontreated control group (Figure 1B). In this experiment, the small nucleolar RNA, *U6*, was chosen as the reference miRNA. In addition, *BCL‐2*, that was a target of miR‐34a, was specified. Consistently, the results showed that the expression level of the *BCL‐2* gene was significantly decreased in the MPP^+^‐treated SH‐SY5Y cells, as compared with the nontreated control group (Figure 1C).

### 3.4 Molecular signaling pathway enrichment analysis on the miR‐34a targetome

**Figure 2:**
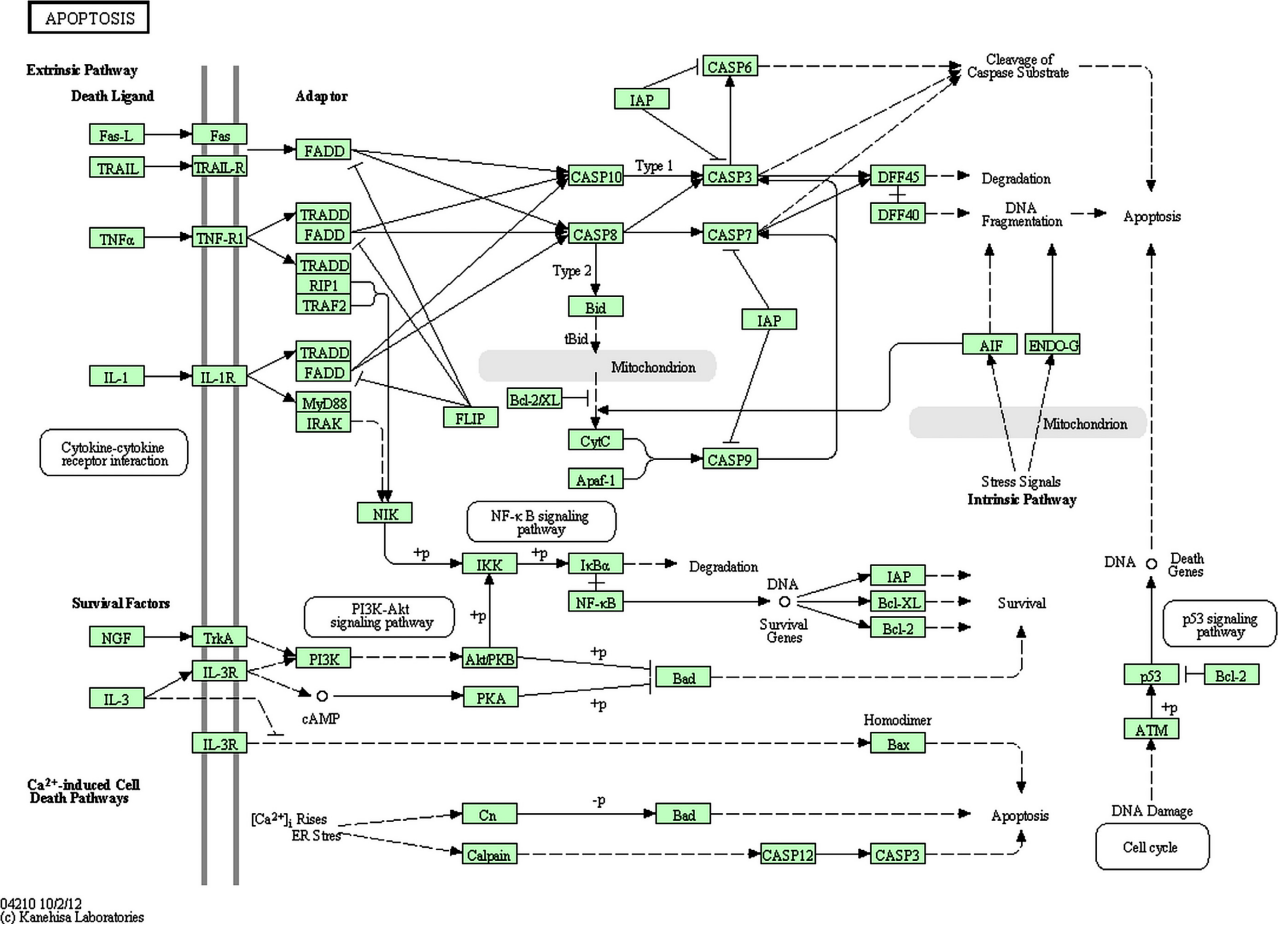
Signaling pathway analysis of KEGG and PANTHER pathways shows that the miR‐34a/*BCL‐2* axis is mostly involved in apoptotic pathways

**Figure 3:**
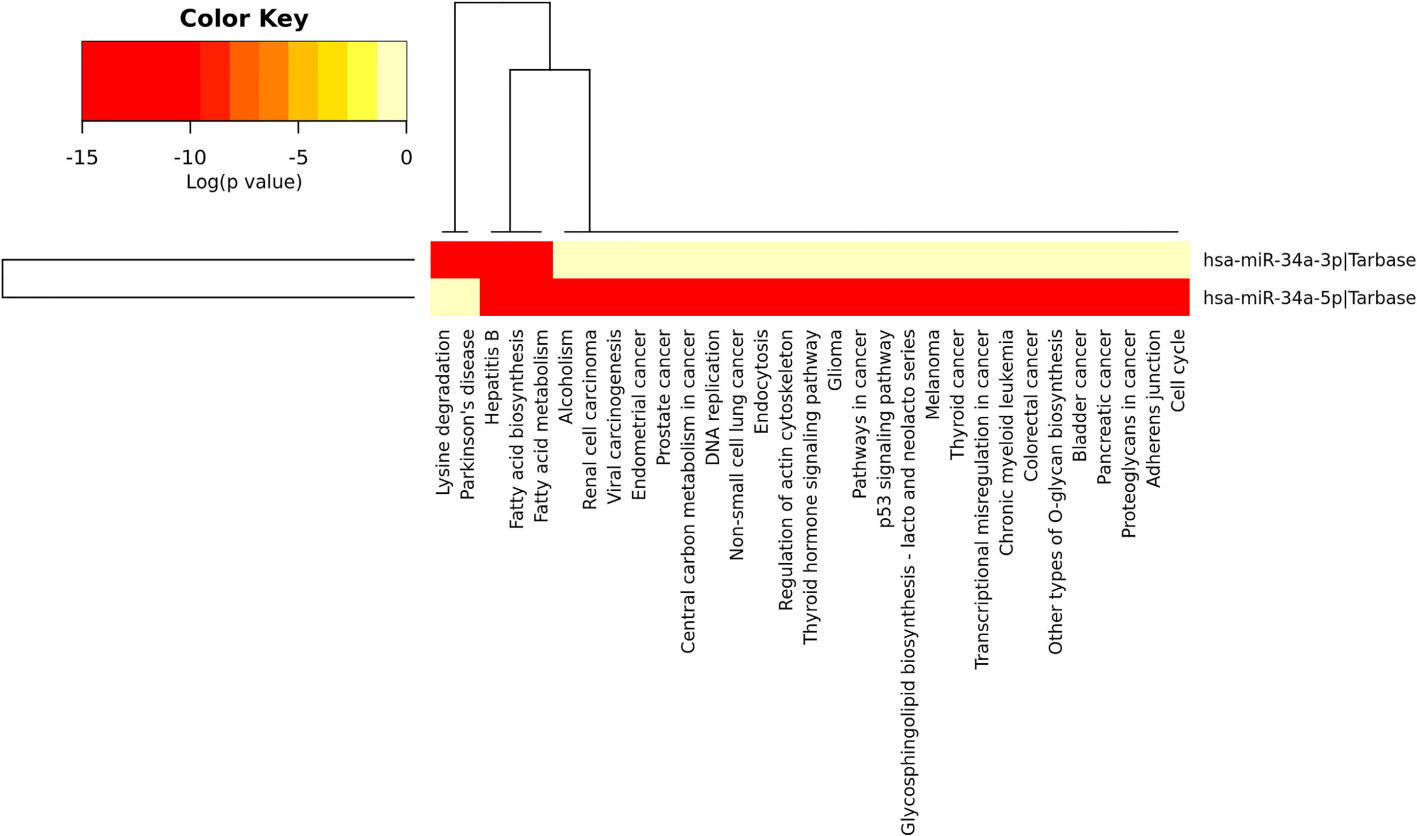
The heatmap view of miR‐34a‐related signaling pathways. The heatmap displays miR‐34a‐related pathways based on DIANA miRPath *v*.3. Here, *p*‐value characterizes the inspected signaling pathways significantly enriched with miR‐34a targets. Also, color gradient indicates pathway importance with red representing the most importance, and the pale yellow as the least one

Data suggested the possible role of miR‐34a in the oxidative stress and apoptosis of SH‐SY5Y cells. Therefore, data collection from the Targetscan database showed that there were about 500 targets for miR‐34a (data not shown). Among all target genes, we wondered whether *BCL‐2* had a significant change as a result of miR‐34a upregulation as various studies had already shown the important role of the BCL‐2 protein family in apoptosis (Pellegrini & Strasser, 2013). miRTarBase database confirmed that *BCL‐2* was a real target gene of miR‐34a. Inputting the *BCL‐2* gene name in KEGG database yielded multiple signaling pathways in which apoptosis was located at the top (Figure 2). Likewise, *BCL‐2* was involved in other pathways including neurotrophin signaling and PI3K‐Akt signaling pathways in KEGG. PANTHER database also confirmed that *BCL‐2* was involved in apoptosis and oxidative stress response signaling pathways (Table 2). Interestingly, using DIANA miRPath *v*.3, we showed that the heatmap of the pathways in which miR‐34a was involved also depicted pathways such as apoptosis, Parkinson's disease, and other ones (Figure 3).

**Table 2:** *BCL‐2* signaling pathways

Signaling pathways	Databases
Apoptosis	KEGG & PANTHER
Neurotrophin signaling	KEGG
PI3K‐Akt signaling pathway	KEGG
Oxidative stress response	PANTHER

## 4 DISCUSSION

Several studies have shown that miRNAs are abundantly found in brain; therefore, any modifications in the brain miRNA network may contribute to the development of neurodegenerative diseases (Hébert & Strooper, 2009). However, it is still not clear how most miRNAs play a role in the disease progression.

Here, we have focused on miR‐34a as different studies have indicated the miR‐34a role in aging‐related disorders including Alzheimer's disease. A study by Chang *et al*. showed that miR‐34a overexpression induced apoptosis in p53^−/−^ cells (Chang et al., 2007). Also, miR‐34a upregulation was indicated in epilepsy, while using antagomir against miR‐34a showed protective effects against the apoptosis of neuronal cells (Hu et al., 2012). Moreover, the upregulation of miR‐34a has been detected in SH‐SY5Y cells induced by 6‐OHDA (Ba et al., 2015).

Our results confirmed previous studies showing the significant upregulation of miR‐34a during the apoptosis of SH‐SY5Y cells.

Various studies have shown the great impact of miR‐34a on *BCL‐2* expression. Studies using the luciferase reporter assay have specified the direct interaction of miR‐34a with 3′ UTR of the *BCL‐*2 gene (Lin et al., 2014). Interestingly, a recent study has shown that the miR‐34a/*BCL‐*2 axis could have a possible role in age‐related disorders including age‐related hearing loss. They have shown that increasing miR‐34a leads to the inhibition of the *BCL‐2* expression and, consequently, promotes apoptosis (Huang et al., 2017). Moreover, Wang et al. (2009 have shown that the miR‐34a expression is inversely correlated with the BCL‐2 protein, and miR‐34a knockdown leads to the increased levels of the BCL‐2 protein in SH‐SY5Y cells. Our results confirmed previous studies indicating the significant downregulation of *BCL‐2* in SH‐SY5Y cells treated with MPP^+^ as compared to our nontreated control group.

Our computational analyses also revealed that pathways such as neurotrophin signaling and PI3K‐Akt signaling pathways, as well as apoptosis and oxidative stress response signaling pathways, were probably the most important ones affected by *BCL‐2* and thus miR‐34a. Consequently, it seems that miR‐34a/*BCL‐2* axis is also important in DA neurons affecting apoptosis in these cells and may contribute to the pathogenesis of PD.

To conclude, the current study indicated that miR‐34a upregulates in MPP^+^‐treated SH‐SY5Y cells, as compared to the nontreated control group. Hence, the therapeutic potential of miR‐34a through inhibiting *BCL‐2* could be considered in order to inhibit PD progression.

## CONFLICT OF INTEREST

None of the authors has any conflict of interests to disclose and all authors support submission to this journal.
