# Supplementary figures and images for: miR‐34a/BCL‐2 signaling axis contributes to apoptosis in MPP+‐induced SH‐SY5Y cells
Source: Mol Genet Genomic Med. 2018 Sep 16;6(6):975–81. doi: 10.1002/mgg3.469 (PMC6305653; doi:10.1002/mgg3.469)

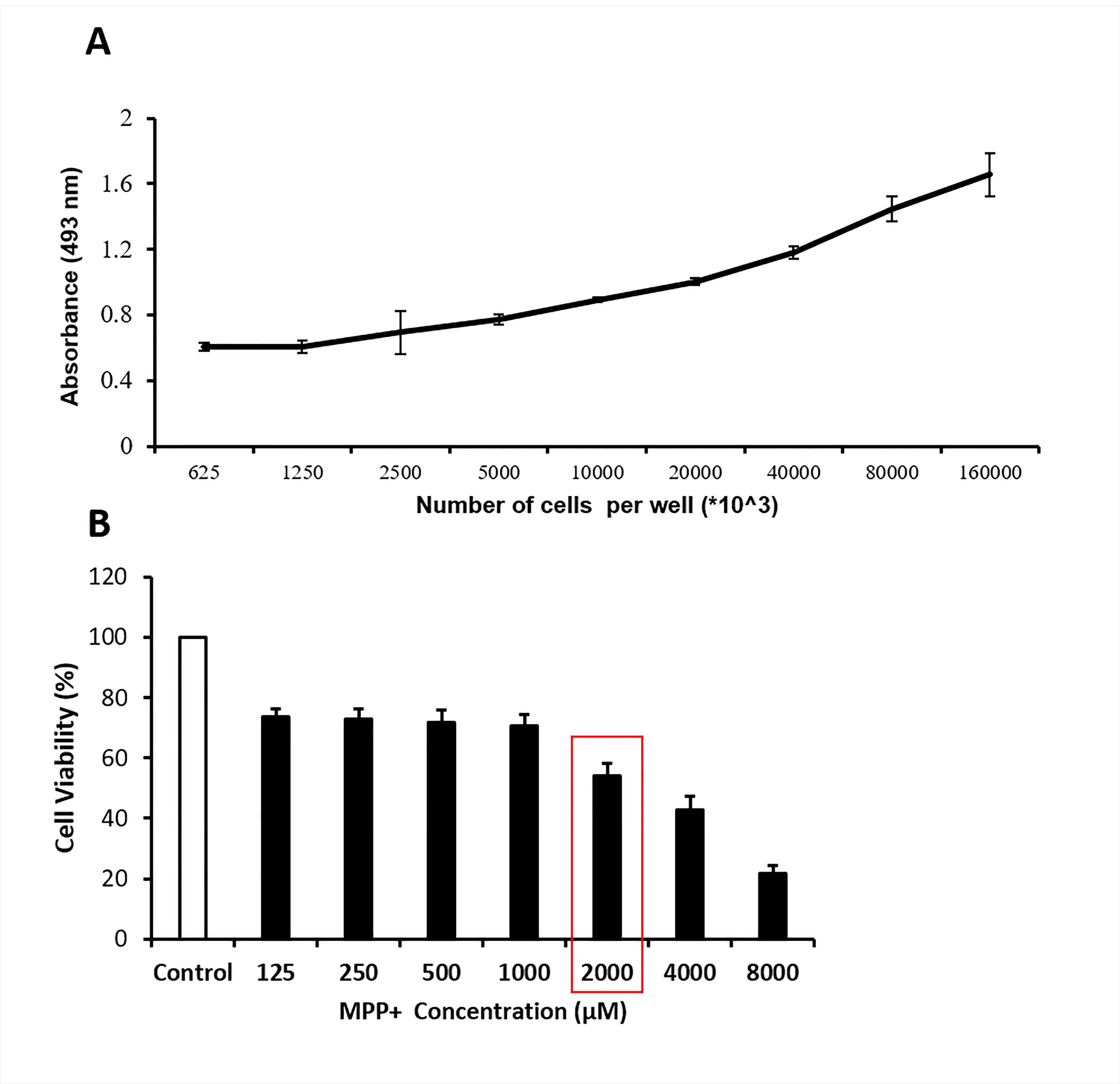

Supplement: Supplementary file 1 [file MGG3-6-975-s001.tif]
